# Genome-Wide Identification and Analysis of APC E3 Ubiquitin Ligase Genes Family in *Triticum aestivum*

**DOI:** 10.3390/genes15030271

**Published:** 2024-02-21

**Authors:** Jinnan Wang, Tianye Zhang, Aizhu Tu, Haoxin Xie, Haichao Hu, Jianping Chen, Jian Yang

**Affiliations:** 1 State Key Laboratory for Managing Biotic and Chemical Threats to the Quality and Safety of Agro-Products, Institute of Plant Virology, Ningbo University, Ningbo 315211, China; 15258141571@163.com (J.W.); ztye1995@163.com (T.Z.); tuuu414@163.com (A.T.); xiehaoxin949@163.com (H.X.); huhdfs@163.com (H.H.); 2 Key Laboratory of Biotechnology in Plant Protection of MARA and Zhejiang Province, Institute of Plant Virology, Ningbo University, Ningbo 315211, China

**Keywords:** wheat, APC E3 ubiquitin ligase, plant immunity, plant gene expression

## Abstract

E3 ubiquitin ligases play a pivotal role in ubiquitination, a crucial post-translational modification process. Anaphase-promoting complex (APC), a large cullin-RING E3 ubiquitin ligase, regulates the unidirectional progression of the cell cycle by ubiquitinating specific target proteins and triggering plant immune responses. Several E3 ubiquitin ligases have been identified owing to advancements in sequencing and annotation of the wheat genome. However, the types and functions of APC E3 ubiquitin ligases in wheat have not been reported. This study identified 14 members of the APC gene family in the wheat genome and divided them into three subgroups (CCS52B, CCS52A, and CDC20) to better understand their functions. Promoter sequence analysis revealed the presence of several cis-acting elements related to hormone and stress responses in the APC E3 ubiquitin ligases in wheat. All identified APC E3 ubiquitin ligase family members were highly expressed in the leaves, and the expression of most genes was induced by the application of methyl jasmonate (MeJA). In addition, the APC gene family in wheat may play a role in plant defense mechanisms. This study comprehensively analyzes APC genes in wheat, laying the groundwork for future research on the function of APC genes in response to viral infections and expanding our understanding of wheat immunity mechanisms.

## 1. Introduction

Post-translational modifications (PTMs) play a crucial role in regulating protein function and protein–protein interactions. PTMs include methylation, acetylation, ubiquitination, phosphorylation, glycosylation, and SUMOylation [1,2]. Ubiquitination is a common PTM that modulates various cellular processes in eukaryotes [3]. It also plays a significant role in plant biology, including abiotic and biotic stresses, as well as immune responses [4,5]. Ubiquitination involves a series of enzymatic cascades involving three key enzymes, E1s, E2s, and E3 ubiquitin ligases. E3 ubiquitin ligases act as receptors that recognize target proteins [6]. A complete ubiquitin E3 ligase-encoding open reading frame library was previously created in rice (*Oryza sativa* L.), which identified seven types of E3 ubiquitin ligases, including RING, U-Box, HECT, F-box, BTB, DWD, and APC (anaphase-promoting complex) [7]. Among these, the RING, U-Box, and HECT E3 ubiquitin ligases have been widely investigated in wheat. However, the function of APC E3 ubiquitin ligases in wheat remains unclear.

APC is an E3-ubiquitin ligase that is conserved in eukaryotes. This enzyme is crucial for regulating cell proliferation and the cell cycle in eukaryotic cells, as it ubiquitinates specific target proteins for degradation by the 26S proteasome [8,9,10]. APC has been extensively studied in both fungal and animal cells because of its significant role in the cell cycle. It targets not only proteins directly related to cell cycle progression, such as cell cycle proteins and securin, but also a wide range of other proteins, including transcription factors and hormone receptors [11]. Functional characterization of APCs has revealed that APC E3-ubiquitin ligases are necessary for gametophyte development and/or embryogenesis [12]. Recent studies have identified a key role for APC in regulating cell differentiation and function in various cell types by employing diverse molecular mechanisms [13]. APC-knockout mutants and transgenic plants overexpressing OSD1 or UVI4 have demonstrated enhanced resistance to the virulent bacterial pathogen *Pseudomonas syringae pv tomato* DC3000, suggesting that deregulation of the cell cycle affects the expression of plant immune-related genes, including *R* genes [14]. APC modulates the function of cell cycle-related proteins, leading to a defense response [15]. In addition, studies have shown that APC can interact with nine effectors of bacterial and oomycete pathogens, and several *Arabidopsis* proteins that may be involved in defense responses. Interactome analysis of *Arabidopsis* proteins with effectors of bacteria and oomycetes further supports the idea that the cell cycle is a battlefield between plants and pathogens [16].

Wheat is a globally significant food crop [17]. However, it is susceptible to viral infections that can lead to significant economic losses in agriculture [18]. One such virus is Chinese wheat mosaic virus (CWMV), which is a member of the genus Furanovirus and is primarily transmitted by *Polymyxa graminis*, a specialized soil-borne organism [19]. CWMV can cause yield reductions of 10–30% in wheat fields [20] and has become increasingly prevalent in winter wheat planting regions in recent years [21]. CWMV is an RNA virus with two positive-sense single-stranded RNAs, RNA1 and RNA2. RNA1 contains three open reading frames (ORFs), each encoding three proteins. ORF1 encodes the methyltransferase (MET). ORF2 encodes an RNA-dependent RNA polymerase (RDRP). ORF3 encodes a movement protein (MP). RNA2 contains four proteins: two minor CP-related proteins (N-CP and CP-RT), a major capsid protein (CP), and a cysteine-rich protein (CRP) [20].

In this study, we conducted a comprehensive analysis of the APC E3 ubiquitin ligases present in the wheat genome. We identified 14 APC E3 ubiquitin ligases and examined their phylogenetic relationships and evolutionary patterns. We identified several hormone-related cis-acting regulatory elements. We analyzed the expression levels of APC E3 ubiquitin ligases in various tissues at different temperatures and in response to methyl jasmonate (MeJA) and CWMV. This study aimed to provide a reliable bioinformatics foundation for future research on the APC E3 ubiquitin ligase family, particularly in relation to its association with viral infections.

## 2. Materials and Methods

### 2.1. Identification of APC Genes Family in Wheat

Five reported rice protein sequences (OsCDC20-1, OsCDC20-2, OsCDC20-3, OsCCS52A1/TE/TAD1, and OsCCS52B) were obtained from the NCBI database (https://www.ncbi.nlm.nih.gov/, accessed on 15 October 2023). Using Pfam (http://pfam-legacy.xfam.org/, accessed on 15 October 2023), it was confirmed that all five rice proteins contain a conserved domain, WD40 [22]. To search for potential APC E3 ubiquitin ligase homologs in the wheat database, the rice protein sequences were submitted to the Ensembl Plant database (http://plants.ensembl.org/Triticum_aestivum/Info/Index, accessed on 15 October 2023) using the Protein Basic Local Alignment Search Tool (BLASTP) [23]. APC E3 ubiquitin ligase homologs in wheat were screened using a stringency of <1 × 10−6 and ID% > 50 as the cut off. The Conserved Domain Database (CDD, https://www.ncbi.nlm.nih.gov/cdd, accessed on 15 October 2023) was used to identify potential APC E3 ubiquitin ligases in wheat, excluding protein sequences lacking the WD40 structural domain [24]. Fourteen APC genes were identified in wheat (Supplementary Table S1).

### 2.2. Physicochemical Properties of APC Genes in Wheat

The ProtParam tool (https://web.expasy.org/protparam/, accessed on 19 October 2023) was used to acquire physicochemical information related to APC genes in wheat, including the number of amino acids, molecular weight, and isoelectric point [25].

### 2.3. Multiple Sequence Alignment and Phylogenetic Tree Construction

MEGA11 was used for multiple sequence alignment of all the obtained protein sequences and to construct a phylogenetic tree of APC genes in different species [26]. The neighbor-joining method was used to construct a phylogenetic tree with a bootstrap value set to 1000 and a *p*-value of less than 0.05.

### 2.4. Gene Structure and Motif Analysis of APC Genes in Wheat

GFF3 annotation information for the entire wheat genome was obtained from the Ensembl Plants database, and TBtools was used to visualize the gene structure in wheat. Conserved motifs in APC genes were determined using the MEME online program (http://alternate.MEME-Suite.org/tools/MEME, accessed on 15 October 2023). MEME analysis included the sum of 10 motifs with other default settings [27].

### 2.5. Predicting Protein Structure of APC Genes in Wheat

The biological functions of proteins are largely dependent on their tertiary structure, which is determined by their amino acid sequences during folding. Tertiary structures of the APC-type E3 ubiquitin ligase in wheat were generated using the SWISS-MODEL homology modeling server (https://swissmodel.expasy.org/, accessed on 15 October 2023). The homology of the 14 proteins was modeled based on GMQE [28]. The TaCCS52B subgroup was modeled in 3D using the AlphaFold DB model of Q2R1T0_ORYSJ. The TaCCS52A subgroup was modeled using the AlphaFold DB model of J3LJF2_ORYBR. The TaCDC20 subgroup was modeled in 3D using the AlphaFold DB model of C0PLV0_MAIZE.

### 2.6. Chromosome Localization and Gene Duplication

To investigate the distribution of genes on wheat chromosomes and gene duplication events, reference information on the wheat genome was obtained from the Ensembl Plant Database. TBtools was used to analyze chromosomal localization and gene duplication events in the wheat genome [29].

### 2.7. Prediction of Cis-Acting Elements in the Promoter

To identify potential cis-acting elements in the promoter sequences, we downloaded 14 APC genes upstream of 2000 bp sequences. PlantCare database was used to predict potential cis-acting elements, and TBtools was used for visualization [30].

### 2.8. Plant Materials, Growth Conditions, and Viral Inoculation

The YM158 strain used in this study was provided by Dr. Jian Yang. The YM 158 plant was cultivated in an artificial greenhouse at 25 °C with a 16 h: 8 h, light–dark photoperiod. Wheat plants were subjected to stress treatment when they reached the three-leaf stage. For the temperature stress treatment, the plants were placed in growth cabinets at different temperatures (8, 15, and 25 °C) with a 16 h: 8 h, light: dark photoperiod, with the 8 °C-treated wheat serving as the control. The wheat plants were inoculated with CWMV and Barley stripe mosaic (BSMV), as described previously [20].

### 2.9. RNA Isolation and RT-qPCR Analysis

RNA was extracted from each sample (e.g., root, stem, or leaf) using the HiPure Plant RNA Mini kit (Magen, Guangzhou, China) and then reverse transcribed into cDNA using the First Strand cDNA Synthesis kit (Toyobo, Kita-ku, Osaka, Japan), where 1 μg of total RNA was added to 20 μL of the reaction system [31]. The real-time fluorescence quantitative PCR (RT-qPCR) was detected using the SYBR Green RT-qPCR mixture (Vazyme, Nanjing, China) on an ABI7900HT sequence detection system (Applied Biosystems, Foster City, CA, USA) [31]. The 2^−ΔΔCt^ method was used to quantify the relative gene expression [31]. The *T. aestivum* cell division cycle (TaCDC) (Accession number: XM_020313450) was used as an internal reference gene [20,32]. At least three independent biological replicates were used for each treatment group. Primers were designed using the NCBI Primer-BLAST. Primers used in this study are listed in Supplementary Table S2.

### 2.10. Analysis of APC Gene Expression in Wheat under MeJA Treatment

To evaluate the effect of hormones on APC gene expression in wheat, three genes were randomly selected from each subgroup and treated with 100 μM MeJA at the three-leaf stage. Wheat plants treated with distilled water served as a negative control [33]. Three biological samples were collected separately at four different time points (0, 2, 4, and 8 h) for total RNA extraction. The expression levels of APC genes were analyzed using RT-qPCR following hormone treatment.

### 2.11. Expression Profiling of APC Genes in Different Wheat Tissues

To analyze the expression levels of the three genes in various tissues, RT-qPCR was used to detect gene expression levels in different tissues, including TL (top leaf), ML (middle leaf), BL (bottom leaf), ST (stem), and RT (root). Three biological samples were collected from each tissue and the expression levels were determined accordingly.

### 2.12. BSMV-Induced Gene Silencing

PCR was used to obtain specific gene fragments containing *Not I* and *Pac I* restriction sites. In vitro transcripts of BSMV RNA α, RNA β, and RNA γ were prepared from linearized plasmid DNAs (pBSMV-α, pBSMV-β, and pBSMV-γ) using the Message T7 in vitro transcription kit (Ambion, Austin, TX; Promega, Shenzhen, China) following the manufacturer’s instructions. The second leaf of YM158(S) at the two-leaf stage was infected with BSMV (BSMV: *γ*, BSMV: *TaCCS52B-2*, BSMA: *TaCDC20-3*) [31]. The third leaf of each plant was inoculated with CWMV at seven days post-inoculation (dpi).

## 3. Results

### 3.1. Identification and Analysis of APC Genes Family in T. aestivum

**Figure 1:**
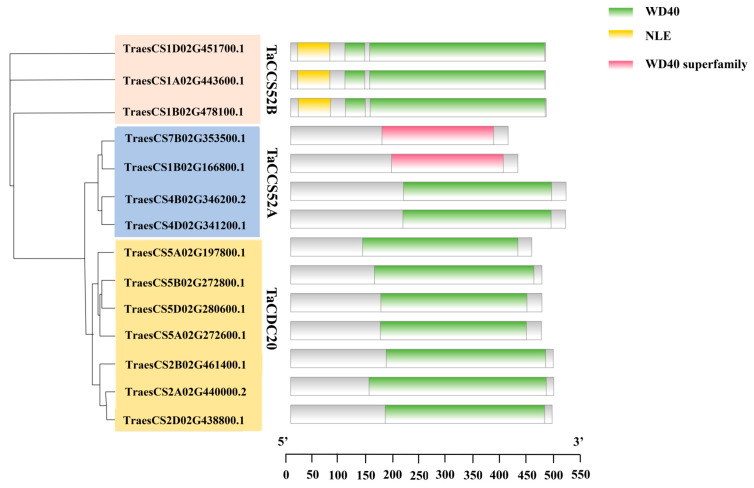
Phylogenetic relationship and conserved domain analysis of each APC genes subgroup in wheat. The conserved domains of the APC gene family are represented on the right, where the green rectangles represent the WD40 conserved domains, and pink and yellow represent the WD40 superfamily and NLE conserved domains, respectively. The left side of the figure represents the division of the 14 APC genes into three subgroups, TaCDC20, TaCCS52A, and TaCCS52B, based on their conserved structural domains and phylogenetic tree, and are highlighted in different colors.

In this study, we performed a genome-wide analysis to identify members of the APC family in the wheat genome based on five APC genes in rice [7]. The APC protein sequences in rice were used as query sequences for BLASTP searches of the wheat genome. Based on these analyses, we identified 14 genes with more than 50% similarity between wheat and rice and found that paralogous and orthologous events occurred during the evolutionary process of APC genes in wheat. Then, we categorized the 14 genes into three subgroups, namely, TaCDC20, TaCCS52A, and TaCCS52B, based on their phylogenetic relationship and functional similarity to previously characterized APC E3 ubiquitin ligases in rice (Figure 1) [7]. Prediction of conserved domains in these proteins revealed the presence of the WD40 domain in all the analyzed proteins. In addition, members of the TaCCS52B subgroup had an NLE structural domain. The presence of diverse conserved domains suggests that APC E3 ubiquitin ligases in wheat perform different biological functions (Supplementary Table S5). Analysis of the physicochemical properties of these genes revealed that the molecular weight of APC genes in wheat ranged from 43.51 to 54.73 kDa, with an isoelectric point of 7.22 to 9.29 and a protein length of 405 to 513 aa (Table 1).

### 3.2. Phylogenetic Tree Analysis of APC Genes in Different Species

**Figure 2:**
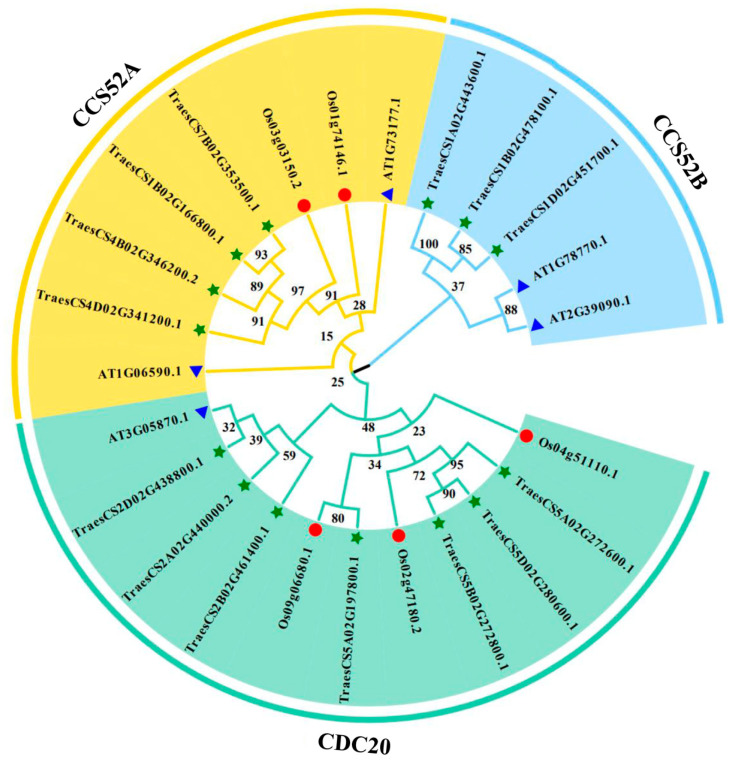
Phylogenetic tree of APC genes in *O. sativa*, *A. thaliana*, and *T. aestivum*. The protein sequences of *O. sativa*, *A. thaliana*, and *T. aestivum* were aligned using MEGA11, and a phylogenetic tree was constructed with 1000 bootstrap values. The 25 APC genes were divided into three groups (CDC20, CCS52A, and CCS52B) and are highlighted in different colors. The blue triangle represents *A. thaliana*, the red circle represents *O. sativa*, and the green star represents *T. aestivum*.

The phylogenetic relationships of APC genes in diverse species were further analyzed by constructing a phylogenetic tree for O. Sativa, *Arabidopsis thaliana*, and T. aestivum (Figure 2; Supplementary Table S1). APC genes were classified into three distinct subgroups. The CDC20, CCS52A, and CCS52B subgroups comprised seven, four, and three wheat species, respectively.

### 3.3. Structure of APC Genes and Motif Analysis in Wheat

**Figure 3:**
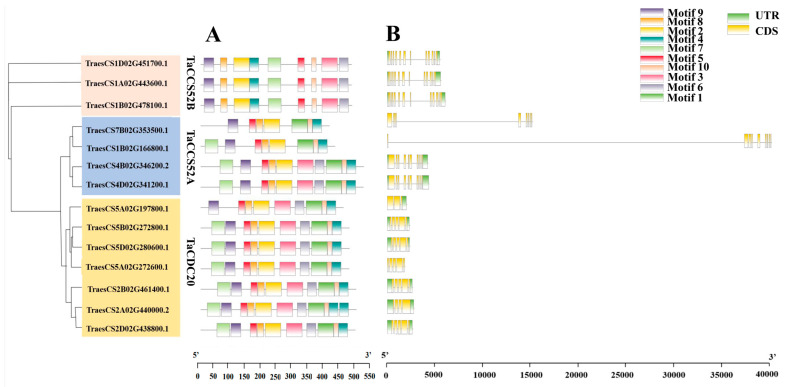
Structural analysis of 14 APC E3 ubiquitin ligases in wheat. (**A**) Motif analysis of 14 APC genes in wheat. M1–M10 motifs are shown in different colored boxes. (**B**) Gene structure analysis of the 14 APC genes in wheat, with yellow rectangular boxes representing coding sequences (CDS) and green rectangular boxes representing the untranslated region (UTR). Sequence lengths are indicated by the scale bar.

The potential functions of the wheat APC genes were analyzed using the MEME online tool, which aided in the identification of possible conserved motifs. As shown in Figure 3A, all genes contained M2, M4, M5, M8, M9, and M10 motifs. In addition, TaCCS52A subgroups had M3, M6, and M7 motifs, whereas TaCDC20 subgroups and TaCCS52A subgroups had M1 motifs (Supplementary Table S6). Notably, these motifs might play important roles in the biological functions of these genes. Further investigation of the structural differences among the APC gene family members in wheat using gDNA sequence analysis revealed that the TaCDC20, TaCCS52A, and TaCCS52B subgroups had 3–5, 8–10, and 12 exons, respectively. These differences indicated that members of this family have a high degree of genetic polymorphism (Figure 3B).

### 3.4. Structural Model of APC Proteins in Wheat

**Figure 4:**
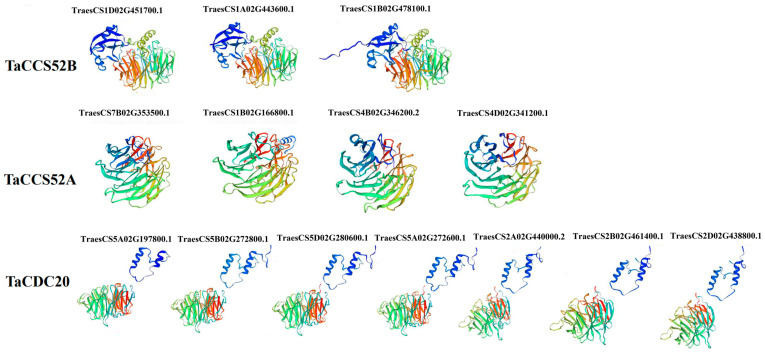
Prediction of the tertiary structure of APC genes in wheat.

To further understand the structural effects of APC proteins in wheat, a three-dimensional (3D) model of the proteins was predicted using SWISS-MODEL, and the optimal model was selected based on GMQE. The results indicated that all genes were suitable for use as models, suggesting the preservation of their structural integrity throughout their evolution, which is crucial for their function. However, the tertiary structure of each subgroup differed significantly, indicating the functional diversity of the APC genes in wheat (Figure 4).

### 3.5. Chromosomal Location and Duplication Events of APC Genes in Wheat

**Figure 5:**
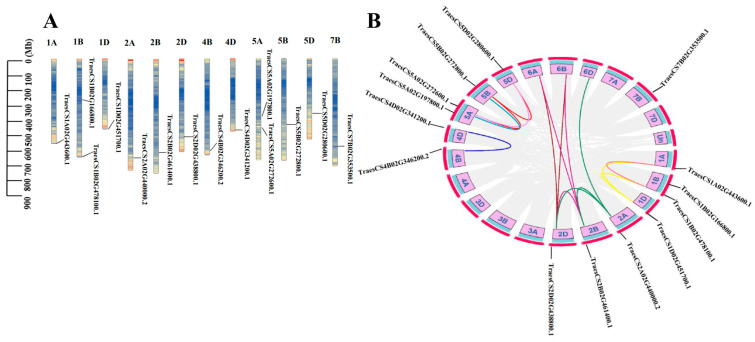
Chromosomal location and duplication events of APC genes in wheat. (**A**) Chromosomal locations of the APC genes in wheat. The names of the chromosomes are located at the top and chromosome lengths are depicted in the scale bar. (**B**) Syntenic analysis of APC family genes in wheat. Chromosomes are represented as a circle and duplication pairs are connected by lines.

The chromosomal locations of individual genes were determined to study the genomic distribution of APC genes in wheat. Chromosomal localization analysis revealed that all 14 APC E3 ubiquitin ligases were uniformly distributed across 12 wheat chromosomes. Specifically, Chr5A and Chr1B contained two genes each, while the remaining chromosomes contained only one gene (Figure 5A; Supplementary Table S3). Collinearity is mainly used to describe the positional relationship of genes on the same chromosome and refers to the distribution or arrangement of homologous genes within and between species. To study the collinearity of APC genes in wheat, TBtools was used to determine the gene duplication relationships. The results indicated that a total of 16 gene pairs of APC-type E3 syntenic paralogs were detected in wheat genome. These results suggest high expansion of the APC gene family in wheat. In addition, five tandem duplication events were observed between the chromosomes, suggesting that tandem chromosomal duplication regions played a significant role in the evolution of the APC family in wheat (Figure 5B; Supplementary Tables S3 and S4).

### 3.6. Prediction and Analysis of Cis-Acting Elements of APC Genes in Wheat

**Figure 6:**
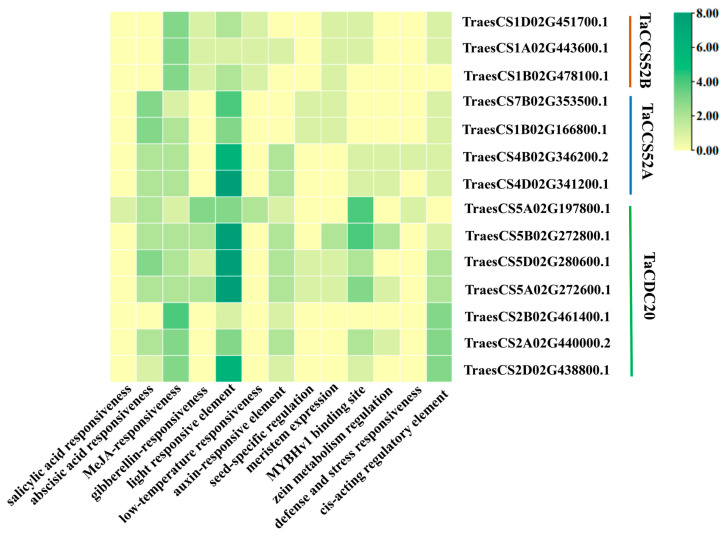
Prediction of cis-acting elements of APC genes in wheat. Target genes are represented on the left, and the names of the cis-acting elements are presented at the bottom of the figure. Average expression values are displayed visually by TBtools. The color scale represents the expression values of each sample. Yellow and green boxes represent the low and high expression levels, respectively.

Analysis of cis-acting elements is crucial for understanding gene regulation and developing transgenic crops. The biological function of APC E3 ubiquitin ligase in wheat was explored based on the prediction of the promoter sequences of the 14 genes using the PlantCARE database. The results revealed the presence of several cis-acting elements in 14 promoters. Notably, cis-acting elements associated with hormone response, such as abscisic acid, MeJA, gibberellin, and salicylic-acid-responsive elements, were relatively abundant. Among these elements, salicylic-acid-responsive elements were present in one member of the TaCDC20 subgroup, abscisic-acid-responsive elements were present in TaCCS52A and TaCDC20, and gibberellin-responsive elements were present in TaCCS52B and TaCDC20. However, MeJA-responsive elements were found in all the subgroups (Figure 6). In addition, several cis-acting elements were bound to MYB transcription factors, and a number of low-temperature response elements were distributed in various subgroups. These findings suggest that hormone levels, abiotic stress, and transcription factors in wheat regulate the expression of APC E3 ubiquitin ligases, which are involved in metabolic processes (Supplementary Table S7).

### 3.7. Tissue Specificity of APC Genes in Wheat

**Figure 7:**
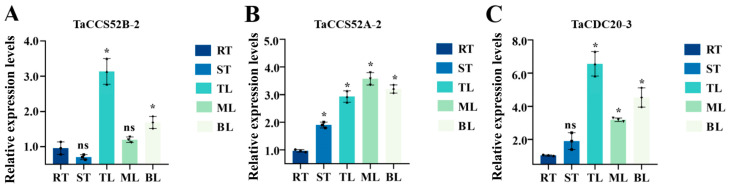
The differential expression of APC genes in different wheat tissues was analyzed by qRT-PCR. (**A**) The differential expression of TaCCS52B-2 in different tissues of wheat. (**B**) The differential expression of TaCCS52A-2 in different tissues of wheat. (**C**) The differential expression of TaCDC20-3 in different tissues of wheat. The plants were divided into five different tissues, with the root as a control. TL, top leaf; ML, middle leaf; BL, bottom leaf; ST, stem; RT, root. Data were collected from three independent biological replicates for each treatment. Data were analyzed using Excel and visualized using GraphPad Prism8. The asterisk (*) indicates statistical significance (*p* < 0.05), and ns indicates that the difference is not statistically significant.

To explore the potential role of APC genes in plant growth and development, one gene from each subgroup was selected and designated *TaCCS52B-2* for *TraesCS1A02G443600.1*, *TaCDC20-3* for *TraesCS5A02G272800.1*, and *TaCCS52A-2* for *TraesCS1B02G166800.1* based on evolutionary analysis. The expression of APC genes in five different tissues (top-, middle-, and bottom-leaf, stem, and root) was analyzed using RT-qPCR. The results indicated the expression of all the three genes in all tissues. *TaCCS52B-2* had relatively higher expression levels in the top and bottom leaves than in the roots, while the expression levels in the stems and middle leaves were not significantly different from those in the roots. The expression of *TaCCS52A-2* was enhanced in all tissues, the expression of *TaCDC20-3* was higher in the leaves than in the roots, and the expression in the stems was not significantly different from that in the roots. These findings suggest that the expression levels of APC E3 ubiquitin ligases in wheat vary according to the tissue type, and that few genes may have important functions at specific stages of wheat development (Figure 7A–C).

### 3.8. Expression Levels of APC Genes in Wheat under Different Stresses

**Figure 8:**
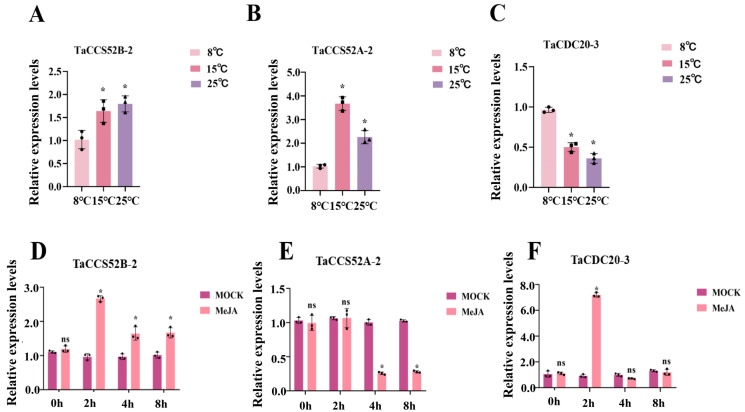
Expression of APC genes under different stresses in wheat. (**A**–**C**) Expression of APC genes in wheat under different temperature treatments. Data were analyzed with Excel and visualized using the GraphPad Prism8 software (Version:8.0.0). (**D**–**F**) Expression of APC genes in wheat after hormone treatment. Three groups of wheat plants under similar growth conditions were sprayed with MeJA or distilled water. The relative expression of the genes after phytohormone treatment was determined using RT-qPCR. Results are representative of a minimum of three biological replicates for each treatment. The asterisk (*) indicates statistical significance (*p* < 0.05), and ns indicates that the difference is not statistically significant.

The cis-acting elements of the genes predicted in the early stages indicated the presence of regulatory elements related to temperature, suggesting that temperature changes could potentially affect gene expression. Accordingly, three groups of wheat plants with similar growth conditions were selected and treated at different temperatures. The expression of *TaCCS52B-2* and *TaCCS52A-2* was enhanced, while that of *TaCDC20-3* was decreased at both 15 °C and 25 °C compared to that at 8 °C. The genes contained cis-acting elements related to temperature regulation, and the different temperatures affected their expression levels (Figure 8A–C).

In cis-acting element analysis, the hormone response elements associated with MeJA were widely distributed in the promoter regions of the genes. To better understand the effects of MeJA on gene expression, the gene expression levels were analyzed for post-hormone treatment. The expression levels of *TaCCS52B-2* and *TaCDC20-3* were significantly increased, while that of *TaCCS52A-2* was decreased in MeJA-treated wheat (2 h) compared to that of control wheat sprayed with distilled water (Figure 8D–F).

### 3.9. Functional Analysis of APC E3 Ubiquitin Ligases in Wheat during CWMV Infestation

**Figure 9:**
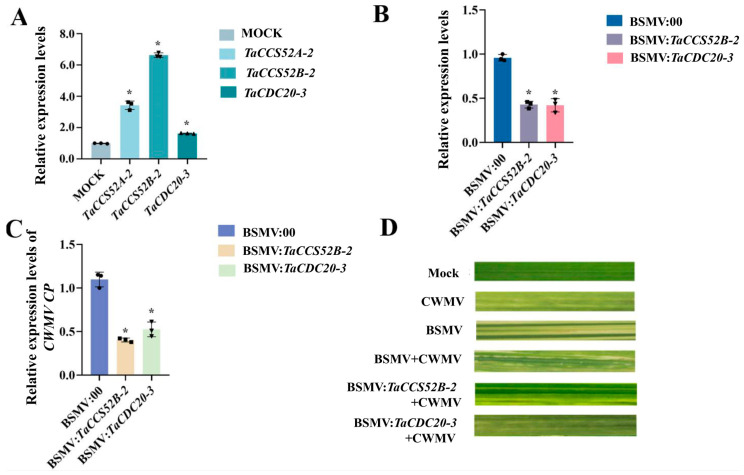
Functional analysis of APC E3 ubiquitin ligases in wheat during CWMV infestation. (**A**) Relative expression of *TaCCS52A-2*, *TaCCS52B-2*, and *TaCDC20-3* in CWMV-inoculated wheat plants analyzed by RT-qPCR using specific primers. (**B**) *TaCCS52B-2* and *TaCDC20-3* were silenced using BSMV-VIGS, and the expression of *TaCCS52B-2* and *TaCDC20-3* transcripts was detected by RT-qPCR. (**C**) Accumulation of CWMV CP after silencing *TaCCS52B-2* and *TaCDC20-3* was analyzed by RT-qPCR. RT-qPCR data were analyzed using the Student’s *t*-test. Results are representative of a minimum of three biological replicates for each treatment (*p* < 0.05). (**D**) Mosaic symptoms in wheat leaves after inoculation with CWMV, BSMV, BSMV+CWMV, BSMV:*TaCCS52B-2*+CWMV, or BSMV:*TaCDC20*+CWMV. MOCK, leaves were inoculated with 1 × Fes buffer as control. The asterisk (*) indicates statistical significance (*p* < 0.05).

To investigate the role of APC genes in wheat immunity, their expression during CWMV infection was analyzed. As shown in Figure 9A, the expression levels of the three selected genes increased significantly during CWMV infection (Supplementary Figure S1).

To further explore the biological functions of APC genes in wheat during CWMV infection, *TaCCS52B-2* and *TaCDC20-3* were silenced using BSMV-VIGS. Subsequently, the transcription levels of *TaCCS52B-2* and *TaCDC20-3* were analyzed in inoculated plants using RT-qPCR (Figure 9B). Further, BSMV:00, BSMV:*TaCCS52B-2*, and BSMV:*TaCDC20-3* were inoculated with CWMV. RT-PCR analysis verified successful infection with BSMV and CWMV in all the co-inoculated plants (Supplementary Figure S2). The relative expression levels of CWMV CP in BSMV:*TaCCS52B-2*+CWMV and BSMV:*TaCDC20-3*+CWMV were significantly lower than those in BSMV:00+CWMV co-inoculated wheat (Figure 9C). All wheat plants infected with BSMV:00+CWMV, BSMV:*TaCCS52B-2*+CWMV, or BSMV:*TaCDC20-3*+CWMV showed mosaic symptoms on the newly formed leaves. Silencing *TaCDC20-3* and *TaCCS52B-2* resulted in milder symptoms than those observed in BSMV:00+CWMV plants (Figure 9D). These findings suggest that silencing of *TaCCS52B-2* and *TaCDC20-3* enhances host resistance to the virus.

## 4. Discussion

APC is a large E3 ubiquitin ligase that regulates cell cycle progression through the degradation of key cell cycle regulators [34]. APC E3 ubiquitin ligases have been identified in various plant species, including O. Sativa, *Rosa chinensis*, and *Zea mays* [35,36,37,38,39]. However, studies related to APC E3 ubiquitin ligases in wheat are limited. With advancements in wheat genome sequencing and annotation, it is now possible to investigate the evolutionary traits and expression of E3 ubiquitin ligases in wheat at the genome-wide level. In this study, we used bioinformatic analysis to identify 14 APC E3 ubiquitin ligases in the wheat genome and classified them into three distinct subgroups based on their predicted gene homology (Figure 1). The genes in each subgroup displayed significant differences in physicochemical properties (Table 1). In the present study, all APC genes in wheat contained at least one of the six conserved motifs (M2, M4, M5, M8, M9, and M10), suggesting a conserved feature among the identified APC genes (Figure 3A). In addition, the conserved domain WD40 was present in all the identified genes (Figure 1). In lower eukaryotes, proteins containing WD40 repeat sequences are mainly associated with growth, the cell cycle, development, and virulence. In contrast, in higher organisms, these proteins play important roles in a variety of cellular functions, such as signal transduction, cell cycle control, intracellular transport, chromatin remodeling, cytoskeleton organization, apoptosis, development, transcriptional regulation, and immune response [40]. Therefore, the APC gene family might be functionally diverse in plants. The number of exons in the identified genes varied widely, ranging from 3 to 12, but was similar within each subgroup. This suggests the presence of conserved exon–intron structures in each subgroup that may be involved in regulating APC gene expression in wheat (Figure 3B). Furthermore, the tertiary structures of all APC E3 ubiquitin ligases in wheat were individually characterized, and large differences were found between the subgroups, indicating diverse functions for these proteins (Figure 4). These results indicated the presence of large sequence variations and diverse biological functions within the APC gene family in wheat. The presence and retention of ancient duplication events in plant genomes, including tandem and segmental replication, has led to the existence of a large number of duplicated genes. These duplicated genes have contributed to the evolution of genes with novel functions, such as increased resistance to diseases and adaptation to different adversities [41,42]. In the present study, homologous gene pairs of wheat APC E3 ubiquitin ligases were not distributed uniformly across wheat chromosomes (Figure 5A). This study identified 16 duplicated gene pairs, including 11 segmental duplication events and 5 tandem duplication events, in the wheat genome, which may contribute to the expansion of the APC gene family in wheat (Figure 5B). A growing number of studies have highlighted the important roles of APC E3 ubiquitin ligases in plant growth and development [43]. In this study, the expression of APC E3 ubiquitin ligases differed among various wheat tissues, but the genes were highly expressed in leaves (Figure 7). These findings suggest that APC E3 ubiquitin ligases are involved in diverse dynamic cellular processes and play important roles in plant growth and development.

Cis-acting regulatory elements are largely responsible for determining the expression patterns of stress-responsive genes [20]. To determine the potential biological function of APC E3 ubiquitin ligases in wheat, cis-acting elements were predicted. The promoter region of APC ubiquitin ligase contained several cis-acting elements associated with abiotic stress, including phytohormones and low-temperature response elements. Phytohormones play a key role in the regulation of biotic and abiotic stresses [43,44,45,46]. MeJA is a phytohormone that is widely present in plants. It can stimulate the expression of plant defense genes and induce chemical defenses when applied exogenously [47]. CUL3BPM E3 ubiquitin ligase regulates the stability of MYC 2, MYC 3, and MYC 4 proteins to modulate the JA signaling pathway [48]. In this study, several MeJA cis-acting elements were identified in the promoter regions of APC ubiquitin ligases in wheat, suggesting that these genes play crucial roles in the MeJA hormone stress response. Therefore, we aimed to explore the relationship between APC genes and MeJA signaling in wheat by verifying the expression levels of these genes post-MeJA treatment. The expression levels of all three selected genes were altered by MeJA treatment. Thus, *TaCCS52A-2*, *TaCCS52B-2*, and *TaCDC20-3* might have potential functions in response to MeJA (Figure 8D–F). Further studies are required to determine the involvement of these genes in the MeJA pathway. Some studies have shown that low temperatures are the major abiotic factors affecting wheat growth [49,50]. Our research aimed to investigate the potential involvement of selected genes in response to low-temperature signals in wheat. The expression levels of the three selected genes varied at different temperatures. Specifically, the expression level of *TaCDC20-3* was downregulated at 15 °C and 25 °C, suggesting that low temperatures enhanced its expression. Overall, the differences in the expression levels of these genes under different temperature treatments suggest that genes in different subgroups have distinct roles at different temperatures (Figure 8A–C).

Plant pathogens cause serious diseases that affect both natural habitats and agricultural environments, thereby threatening plant biodiversity and global food security. Host-adapted microbial pathogens use various infection strategies to evade or counteract plant immunity and to establish replicative ecological niches. Evasion of plant immunity by different pathogens through the suppression of host recognition or immune signals is an important infection strategy for disease causation and a major obstacle to the effective use of host genetic resistance genes for sustainable disease control [51]. Previous studies have shown that changes in APC function in *A. thaliana* induce an immune response [14], while inhibition of NbCdc27B in *Nicotiana benthamiana* plants triggers a defense response and enhances resistance to *Colletotrichum lagenarium* [15]. Plant defense against biotrophic pathogens is associated with programmed cell death (PCD) in infected cells [52]. Previous studies have demonstrated that an APC inhibitor that regulates cell cycle progression can promote PCD by upregulating the expression of NB-LRR genes [14]. Plant viruses, as obligate pathogens, depend on host–plant mechanisms for their life cycle [53]. However, the relationship between APC genes and viruses has not been explored. To investigate whether APC genes in wheat play a role in plant immunity, we analyzed the changes in gene expression after inoculation with CWMV. The expression levels of *TaCCS52A-2*, *TaCCS52B-2*, and *TaCDC20-3* were significantly increased post-CWMV infection (Figure 9A). Based on these findings, we hypothesized that these genes play crucial roles in CWMV infection in wheat. To validate this hypothesis, *TaCDC20-3* and *TaCCS52B-2* were silenced in wheat using VIGS. The results showed that gene-silenced wheat plants inhibited CWMV infection (Figure 9B,C). This study comprehensively analyzed APC genes in wheat and provides a foundation for future research on APC gene function related to wheat response to viral infection.

CWMV is one of the most important pathogens causing wheat mosaic disease in China, which usually causes 10–30% yield loss [20]. So far, the best countermeasure to control this disease is to develop resistant wheat varieties. However, the resistance genes to CWMV infection in wheat remain unidentified. This study comprehensively analyzes APC genes in wheat, laying the groundwork for future research on the function of APC genes in response to viral infections and expanding our understanding of immune mechanisms in wheat. In addition, this study will help us to cultivate new resistant wheat varieties, which will improve agricultural productivity.

## 5. Conclusions

Ubiquitination is an important post-translational modification that plays a significant role in the regulation of endogenous protein stability, enzyme activity, and protein interactions. Ubiquitination is tightly regulated by E3 ligases, and several types of E3 ubiquitin ligases have been identified in wheat. APC, a multifunctional E3 ubiquitin ligase, targets different substrates for ubiquitination and regulates various cellular processes. APC-type E3 ubiquitin ligases have been shown to affect plant immunity. Therefore, we aimed to identify and characterize APC-type E3 ubiquitin ligases in wheat. A total of 14 APC genes were identified in wheat, which were classified into three subgroups, and analyzed for their gene structure and conserved structural domains. All APC genes were highly conserved during evolution. Prediction of their tertiary structures revealed that each subfamily had a unique structure. The promoter regions of all the genes contained hormone-, temperature-, and growth/development-related regulatory elements. The expression of APC genes in different tissues was characterized to decipher their biological functions. All APC family members were highly expressed in the leaves. Most APC family members were induced by MeJA. This study deciphered the potential role of APC genes in plant resistance to viral infection, thereby widening the available knowledge related to the molecular mechanisms underlying wheat–CWMV interactions and contributing to our understanding of potential strategies for wheat resistance to virus infection. Thus, these findings may have broader implications for agricultural practices and lay the foundation for further investigations in this area.

## Figures and Tables

**Table 1:** Basic information of 14 APC proteins in wheat.

Group	Gene ID	Exons	Gene Location	CDS Length (bp)	Size (aa)	MW (kDa)	PI
**TaCDC20**	TraesCS2A02G440000.2	4	2A:690927389-690930130	2427	490	54134.09	8.23
**TaCDC20**	TraesCS2B02G461400.1	5	2B:655009903-655012488	2169	489	53906.73	8.81
**TaCDC20**	TraesCS2D02G438800.1	5	2D:548080286-548082886	2189	487	53485.13	8.80
**TaCDC20**	TraesCS5A02G197800.1	3	5A:401758452-401763369	1782	449	50176.22	7.98
**TaCDC20**	TraesCS5A02G272600.1	5	5A:482315663-482317456	1404	467	51151.27	7.22
**TaCDC20**	TraesCS5B02G272800.1	5	5B:458385818-458388103	1907	468	51338.56	7.23
**TaCDC20**	TraesCS5D02G280600.1	5	5D:381978841-381981151	1939	468	51342.49	7.68
**TaCCS52A**	TraesCS4B02G346200.2	10	4B:640006920-640011111	2178	513	54725.21	9.29
**TaCCS52A**	TraesCS4D02G341200.1	10	4D:498031682-498035987	2304	512	54685.17	9.29
**TaCCS52A**	TraesCS7B02G353500.1	8	7B:611443510-611458578	1218	405	43507.27	9.24
**TaCCS52A**	TraesCS1B02G166800.1	9	1B:294926224-294966206	1272	423	46023.17	8.91
**TaCCS52B**	TraesCS1D02G451700.1	12	1D:493705055-493710507	1799	475	52278.23	8.03
**TaCCS52B**	TraesCS1A02G443600.1	12	1A:591948406-591953951	2037	475	52280.25	8.29
**TaCCS52B**	TraesCS1B02G478100.1	12	1B:686749388-686755409	1884	476	52349.36	8.29

CDS, coding sequence; bp, base pair; aa, amino acid; MW, molecular weight; Da, Dalton; PI, isoelectric point.

## Data Availability

The original contributions presented in the study are included in the article and Supplementary Material, further inquiries can be directed to the corresponding authors.

## Supplementary Materials

The following supporting information can be downloaded at https://www.mdpi.com/article/10.3390/genes15030271/s1. Figure S1: Reverse transcription PCR (RT-PCR) detection of CWMV infection; Figure S2: Detection of CWMV and BSMV by RT-PCR; Table S1: Protein sequences of APC E3 ubiquitin ligases in *O. sativa*, *A. thaliana*, and *T. aestivum*; Table S2: Specific primers used for this article; Table S3: Chromosome distribution of APC E3 ubiquitin ligases in wheat; Table S4: Collinear events of APC E3 ubiquitin ligases in wheat; Table S5: Distribution of the conserved domains of 14 APC E3 ubiquitin ligases in wheat; Table S6: Motif analysis of 14 APC E3 ubiquitin ligases in wheat; Table S7: Cis-acting elements prediction of 14 APC E3 ubiquitin ligases in wheat.
