# Supplementary material for: Genome-Wide Identification and Analysis of APC E3 Ubiquitin Ligase Genes Family in Triticum aestivum
Source: Genes (Basel). 2024 Feb 21;15(3):271. doi: 10.3390/genes15030271 (PMC10970508; doi:10.3390/genes15030271)
Supplement: Supplementary file 1 [file genes-15-00271-s001.zip › supplementary Figures.pdf]

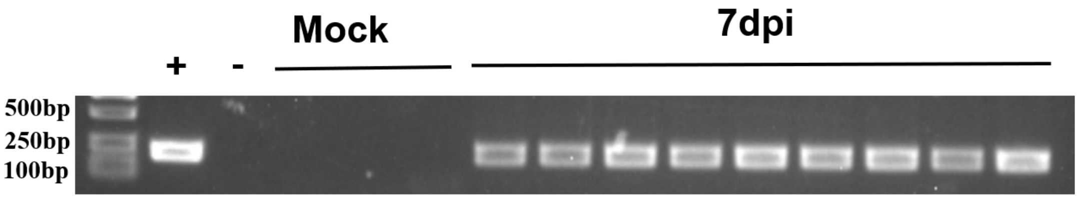

Figure S1. Reverse transcription PCR (RT-PCR) detection of CWMV infection. Total RNA from healthy wheat plants was used as a negative control (-). CWMV RNA 2 was used as a positive control (+). Three plants were analyzed for each treatment.

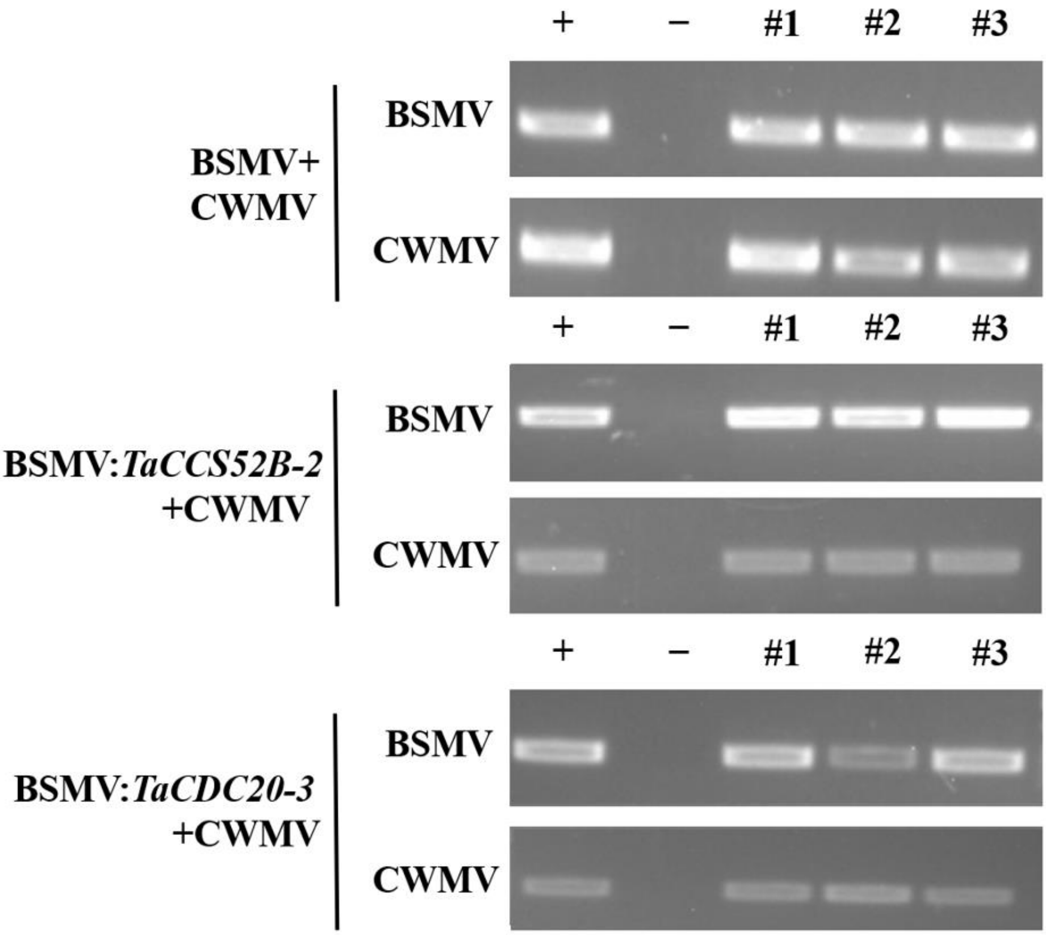

Figure S2. Detection of CWMV and BSMV by RT-PCR. virus-infected and virus-uninfected wheat were used as positive (+) and negative (-) controls, respectively.
